# Genetic and Epigenetic Responses of Autochthonous Grapevine Cultivars from the ‘Epirus’ Region of Greece upon Consecutive Drought Stress

**DOI:** 10.3390/plants13010027

**Published:** 2023-12-21

**Authors:** Grigorios Maniatis, Eleni Tani, Anastasios Katsileros, Evangelia V. Avramidou, Theodora Pitsoli, Efi Sarri, Maria Gerakari, Maria Goufa, Maria Panagoulakou, Konstantina Xipolitaki, Kimon Klouvatos, Stamatia Megariti, Polixeni Pappi, Ioannis E. Papadakis, Penelope J. Bebeli, Aliki Kapazoglou

**Affiliations:** 1 Laboratory of Plant Breeding and Biometry, Department of Crop Science, Agricultural University of Athens, Iera Odos 75, 11855 Athens, Greece; gr_maniatis@yahoo.gr (G.M.); katsileros@aua.gr (A.K.); sarri@aua.gr (E.S.); mgerakari@aua.gr (Μ.G.); marog@aua.gr (M.G.); maria_panagoulakou@yahoo.com (M.P.); kwnaxypol@gmail.com (K.X.); kimonklouvatos@gmail.com (K.K.); stamatia.meg@gmail.com (S.M.); bebeli@aua.gr (P.J.B.); 2 Laboratory of Forest Genetics and Biotechnology, Institute of Mediterranean Forest Ecosystems, Hellenic Agricultural Organization-DIMITRA (ELGO-DIMITRA), Ilisia, 11528 Athens, Greece; aevaggelia@yahoo.com; 3 Department of Vitis, Institute of Olive Tree, Subtropical Crops and Viticulture (IOSV), Hellenic Agricultural Organization-DIMITRA (ELGO-DIMITRA), Lykovrysi, 14123 Athens, Greece; pitsoli@elgo.gr; 4 Laboratory of Plant Virology, Department of Viticulture, Vegetable Crops, Floriculture and Plant Protection, Institute of Olive Tree, Subtropical Crops and Viticulture, Hellenic Agricultural Organization DIMITRA (ELGO-DIMITRA), Kastorias 32A, Mesa Katsampas, 71307 Heraklion, Crete, Greece; pappi@elgo.gr; 5 Laboratory of Pomology, Department of Crop Science, Agricultural University of Athens, Iera Odos 75, 11855 Athens, Greece; papadakis@aua.gr

**Keywords:** abiotic stress, dehydration, grafting, rootstock, transcription factors, epigenetic regulation, miRNA, DNA methylation

## Abstract

Within the framework of preserving and valorizing the rich grapevine germplasm of the Epirus region of Greece, indigenous grapevine (*Vitis vinifera* L.) cultivars were characterized and assessed for their resilience to abiotic stresses in the context of climate change. The cultivars ‘Debina’ and ‘Dichali’ displayed significant differences in their response to drought stress as judged by morpho-physiological analysis, indicating higher drought tolerance for Dichali. Hence, they were selected for further study aiming to identify genetic and epigenetic mechanisms possibly regulating drought adaptability. Specifically, self-rooted and heterografted on ‘Richter 110’ rootstock plants were subjected to two phases of drought with a recovery period in between. Gene expression analysis was performed for two stress-related miRNAs and their target genes: (a) miRNA159 and putative targets, *VvMYB101*, *VvGATA-26-like*, *VvTOPLESS-4-like* and (b) miRNA156 and putative target gene *VvCONSTANS-5*. Overall, grafted plants exhibited a higher drought tolerance than self-rooted plants, suggesting beneficial rootstock–scion interactions. Comparative analysis revealed differential gene expression under repetitive drought stresses between the two cultivars as well as between the self-rooted and grafted plants. ‘Dichali’ exhibited an up-regulation of most of the genes examined, which may be associated with increased tolerance. Nevertheless, the profound down-regulation of *VvTOPLESS-4-like* (a transcriptional co-repressor of transcription factors) upon drought and the concomitant up-regulation of *miRNA159* highlights the importance of this ‘miRNA-target’ module in drought responsiveness. DNA methylation profiling using MSAP analysis revealed differential methylation patterns between the two genotypes in response to drought. Further investigations of gene expression and DNA methylation will contribute to our understanding of the epigenetic mechanisms underlying grapevine tolerance to drought stress.

## 1. Introduction

Grapevine owes its long-term adaptation to climate change to its very high genetic diversity, nevertheless, grapevine is one of the most responsive plants to its surrounding environment. Several studies focus on monitoring the intra- and inter-genetic diversity employing molecular techniques to properly identify, valorize and preserve the wealth of grapevine genetic resources, and adopt selection strategies as well as pre-breeding tools towards abiotic stress resilience [1,2]. Moreover, the emerging need to identify genetic material with high fruit quality and unique wine characteristics has intensified the conservation of ancient grapevine genetic material and indigenous cultivars, not only in gene bank collections but also in field collections. In several cases, special care was taken in relation to their sanitary status [3,4,5,6]. In Greece, during the period 1960–1980, intense cultivation of only specific Greek cultivars and the dominant foreign ones resulted in a drastic limitation of the cultivation and under-utilization of the many autochthonous cultivars, leading to significant genetic erosion of biodiversity [7]. In recent years, however, a plethora of research projects have been conducted worldwide to explore and re-utilize the biodiversity of the grapevine autochthonous cultivars [3,4,5,6]. Moreover, maintaining a wide range of cultivars is of fundamental importance for identifying and preserving the most adequate plant genetic material with resilience to specific environmental stressors [8].

Most of the global wine regions are situated in temperate zones, often displaying a Mediterranean climate, which is known for its warm and arid summers. In these areas, grapevines frequently experience bouts of drought, unless deliberate irrigation is employed. Interestingly, a significant portion of the world’s winegrape cultivation currently occurs without irrigation. Recent research suggests that the impact of climate change will exacerbate drought occurrences in these traditional wine-producing areas, necessitating increased irrigation [9]. As concerns persist about the sustainability of irrigation practices, there is a notable emphasis on comprehending the variations in drought tolerance among existing grapevine cultivars [10]. Water use efficiency (WUE) refers to the quantification of carbon assimilation—as biomass or yield—relative to the quantity of water used by the crop. Understanding how plants respond to a shifting climate marked by fluctuations in temperature, precipitation and carbon dioxide (CO_2_) levels, which collectively impact their WUE, is of paramount importance [11].

Grafting, being an ancient agricultural technique, was initially employed to enhance the agricultural attributes of fruits; however, European grapevines commonly undergo grafting onto interspecific hybrid rootstocks to effectively combat phylloxera infestations (*Daktulosphaira vitifoliae*) [12]. Certain rootstocks confer a capacity for drought tolerance [13,14,15]. Consequently, the management of rootstocks holds significant promise as a strategy to augment the resilience of grapevines in the face of water scarcity [15,16].

The regulation of gene expression at the transcriptional level determines the characteristics of a series of processes related to growth, development and adaptability to environmental changes. A dominant role in this process is played by the families of transcription factors (TFs), which govern the expression of other downstream target genes by forming specific protein complexes driving transcriptional processes [17]. Therefore, TFs constitute extremely important regulators of plant growth, development, stress-related responses and environmental adaptability and would serve as valuable tools for plant breeding interventions [18,19]. In this study, we focused on the expression of genes encoding MYB-, GATA- TOPLESS-, CONSTANS-transcription factors and their potential miRNA regulators, miR159 and mir156. Genes belonging to the *MYB* family regulate the function of antioxidant enzymes that lead to the inhibition of reactive oxygen species (ROS) accumulation resulting in increased resilience to stress [20]. Increasing the concentration of antioxidant enzymes is probably an element of a successful response to drought stress conditions [21]. The *GATA* gene family is associated with the regulation of several factors that control plant growth including phytohormones and activating a series of genes linked to photoperiodicity responses and photosynthetic capacity [22]. The family of TOPLESS (TPL) and their associated proteins (Topless Related Proteins (TRP) are repressors of TFs, implicated in diverse developmental processes and in the response to extrinsic challenges [23]. They act as suppressors of hormonal-mediated pathways such as auxin, jasmonic acid, strigolactone and brassinosteroid signal transduction cascades, which are critical for growth and development [23]. The importance of regulating phytohormone-mediated pathways highlights the involvement of these genes in plant growth under normal conditions, but especially under stress [23]. Finally, the CONSTANS family is one of the key TFs that regulate the production of Flower Locus T, which causes floral differentiation, regulating the time and characteristics of flowering. CONSTANS perform a regulatory role in the photoperiodic path that connects daytime with blooming [24]. 

Epigenetics refers to stable and heritable changes in chromatin architecture that do not involve changes in the underlying DNA sequence but profoundly affect gene expression and cellular function ultimately impacting plant phenotype. Alterations in chromatin structure are established by epigenetic mechanisms such as DNA methylation, post-translational histone modification and the action of non-coding RNA molecules such as small interfering RNAs-siRNAs and micro RNAs-miRNAs [25,26]. Epigenetic regulation plays a crucial role in all aspects of plant development as well as in resilience to external stresses. Therefore, epigenetic regulation constitutes a major driver of plant adaptability to environmental challenges [27,28,29].

DNA methylation/demethylation is associated with the response to abiotic stressors and stress tolerance in various crops like cereals, vegetables and woody perennials [26,29]. Drought-induced variations in DNA methylation patterns were reported in economically important crops including rice, barley and mulberry [30,31,32,33,34,35]. Notably, associations of DNA methylation changes with drought-stress memory were reported in some cases [34,36,37]. Nevertheless, although important investigations on methylation landscapes in grapevine clones or cultivars grown in different environments were reported [38,39,40], studies on DNA methylation in grapevines under drought stress are limited [41].

MiRNAs comprise a class of small (20- to 24-nucleotide) non-coding RNAs, involved in the post-transcriptional regulation of target genes through either degradation of the targeted messenger RNAs or translational inhibition. A wide range of miRNAs are associated with plant developmental processes and responsiveness to abiotic stress in a cultivar of plant species [42,43,44]. Under drought stress, numerous miRNAs including *miR159* and *miR156*, were reported to be up-regulated or down-regulated in order to modulate the expression of drought-responsive genes and activate drought-associated biochemical pathways [43,45,46]. 

MiR159 is an ancient, ubiquitous miRNA involved in vegetative, reproductive, seed and fruit development as well as drought and other abiotic stress responses [46,47]. MiR159 mainly targets genes encoding members of the MYB transcription factors family leading to their transcriptional repression and the activation of drought-responsive ABA-mediated pathways [48,49]. Arabidopsis *miR159ab* mutant lines displayed smaller-sized stomata, a decreased number of open stomata and higher survival rates under drought stress as compared to wild-type plants, suggesting that *miR159* down-regulation leads to drought tolerance. Further analysis indicated that miR159 plays a major role in drought stress through a miR159–MYB33–ABI5 regulatory network [50].

MiRNA156 is a well-conserved plant microRNA with vital roles in plant architecture, vegetative and reproductive development as well as abiotic stress responsiveness. It was reported to play a crucial role in conferring tolerance to drought, increased salinity and heat [43]. Alfalfa genotypes overexpressing *miR156* were found to display reduced water loss and increased root growth under dehydration conditions [51,52]. Enhanced drought tolerance mediated by miR156 could be operating through *miR156*-*SQUAMOSA PROMOTR BINDING PROTEIN-LIKE (SPL)* modules [21,52,53,54]. In grapevine, investigations in drought-tolerant and drought-susceptible genotypes showed significant down-regulation of *miR159* in the tolerant genotype (M4) under drought stress but not in the sensitive one, whereas decreased *miR156* transcript abundance was observed in the stressed tissue of both genotypes [13]. Furthermore, in a recent miRNome study, differential expression was revealed for a series of miRNAs upon drought conditions between a stress-tolerant and a stress-susceptible grapevine cultivar. Among those, *miR159c*, *miR156b* and *miR156f* were found to be up-regulated under drought conditions in the susceptible genotype (Cabernet Sauvignon) but not in the tolerant genotype (110 R), suggesting that their expression pattern is associated with drought responsiveness and drought tolerance [49]. Despite the above notable investigations, the response to drought stress of autochthonous grapevine cultivars and its effect on regulatory miRNAs and relevant transcription factor targets has been little studied to date. The main aim of this study was to explore the drought tolerance potential of two indigenous grapevine cultivars, Debina and Dichali, from the region of Epirus, Greece, as well as to evaluate the impact of grafting in the response to drought stress. The specific objectives were to assess the drought response at the level of (a) physiology and (b) gene expression of drought-responsive miRNAs, miR156 and miR159, as well as of potential target genes encoding transcription factors MYB, TOPLESS, GATA and CONSTANS, in self-rooted and grafted grapevine plants. The TF gene families mentioned above include genes of major importance in plant growth and development whose up- or down-regulation depends on environmental conditions. Consequently, there is a significant association between the expression of these genes and the intensifying effects of climate variability and investigating their transcriptional response acquires additional value in the context of ongoing climate change. 

## 2. Results

### 2.1. Drought Stress and Stem Elongation Evaluation

**Figure 1:**
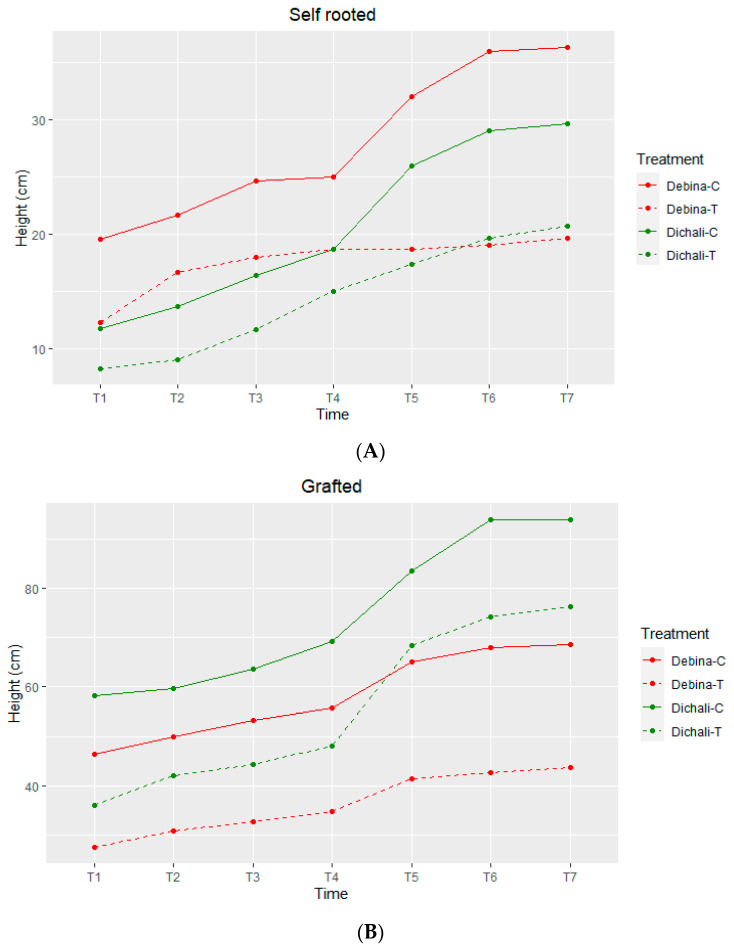
Stem elongation measurements during the experimental procedure in Debina and Dichali in (**A**) self-rooted and (**Β**) grafted plants. C, control; T, treatment.

After subjecting several autochthonous grapevine cultivars of the Epirus region to two phases of drought stress (irrigation with 50% and 25% of water pot capacity) and a recovery period in between, the cultivars ‘Debina’ and ‘Dichali’ were selected for further study due to their contrasting response. More specifically, Debina, a cultivar of major importance for Epirus, exhibited a statistically significant growth rate decline (*p*-value = 0.0035), especially pronounced in self-rooted stressed plants, as compared to control plants (Figure 1A,B). The cultivar Dichali, on the other hand, did not show a decrease in growth rate under water-deficit conditions in heterografts (*p*-value = 0.8385) and only a slight decrease was observed in self-rooted plants (*p*-value = 0.2860). Additionally, an overall statistically significant higher growth rate (*p*-value = 0.0014) was observed in grafted plants as compared to self-rooted plants (Figure 1A,B), regardless of cultivar, which is attributed to the grafting on the Richter 110 rootstock.

### 2.2. Physiological Evaluation

**Figure 2:**
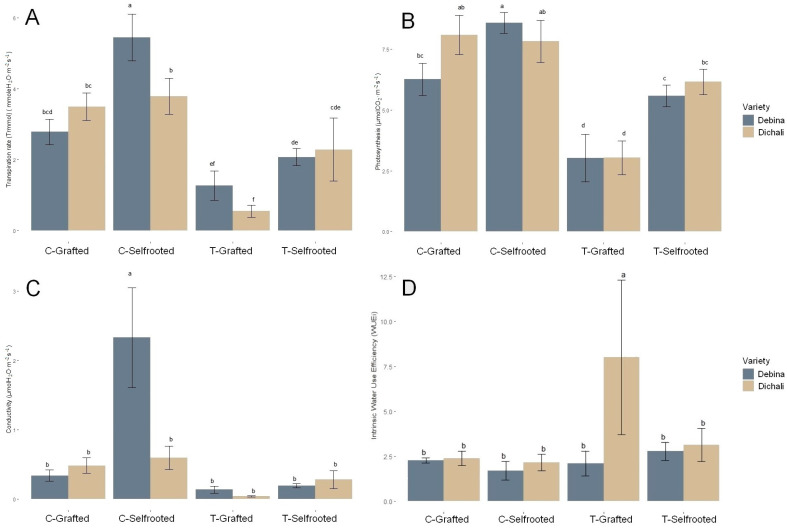
Alterations in the photosynthetic characteristics of the grapevine cultivars Debina and Dichali in response to drought stress upon completion of the experiment. (**A**) Net photosynthetic rate (AN), (**B**) Stomatal conductance (gs), (**C**) Transpiration rate (E), (**D**) Intrinsic Water Use Efficiency (WUEi). C, control plants; T, drought stress treatment. Different lowercase letters indicate differences at a significance level of 0.05.

A decline in the net photosynthetic rate (AN), stomatal conductance (gs) and transpiration rate (Ε) was observed in the stressed plants of both cultivars (Figure 2A–C). On the other hand, an increased WUEi was observed in the grafted water-stressed plants of Dichali (Figure 2D). 

### 2.3. Gene Expression Analysis

Four transcription factor genes with the following annotations were analyzed: *VIT_19s0090g00590*, encoding the putative grape ortholog of Arabidopsis MYB101; *VIT_09s0002g08370*, encoding TOPLESS-RELATED PROTEIN4-like; *VIT_00s0287g00040*, encoding GATA TRANSCRIPTION FACTOR26-like and *VIT_04s0008g07340* encoding zinc finger protein CONSTANS5-like [13], which hereafter will be referred to as: *VvMYB101*, *VvTOPLESS4-like*, *VvGATA26-like* and *VvCONSTANS5-like*, respectively.

**Figure 3:**
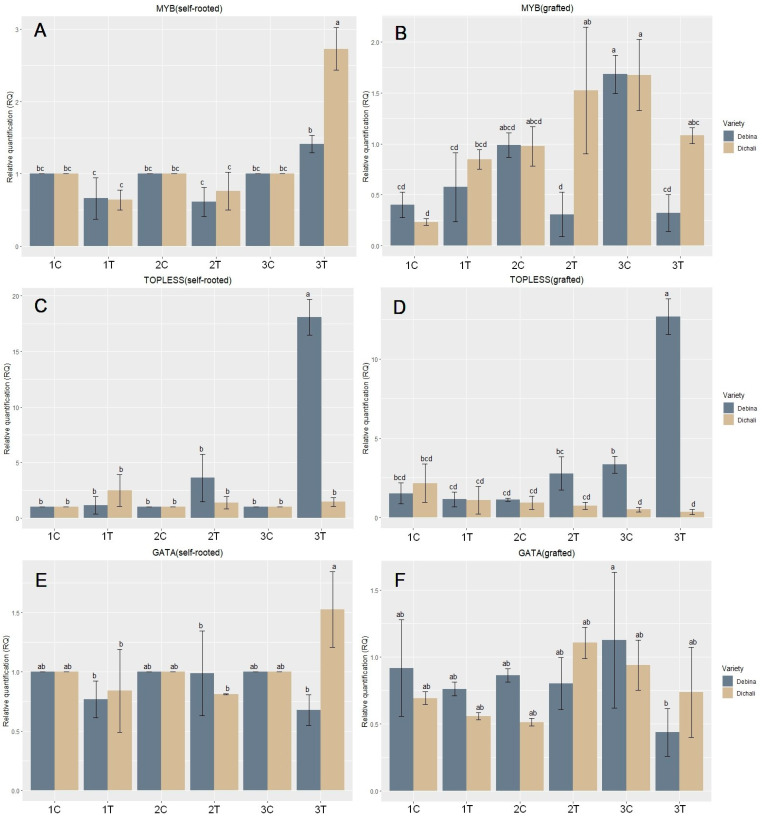

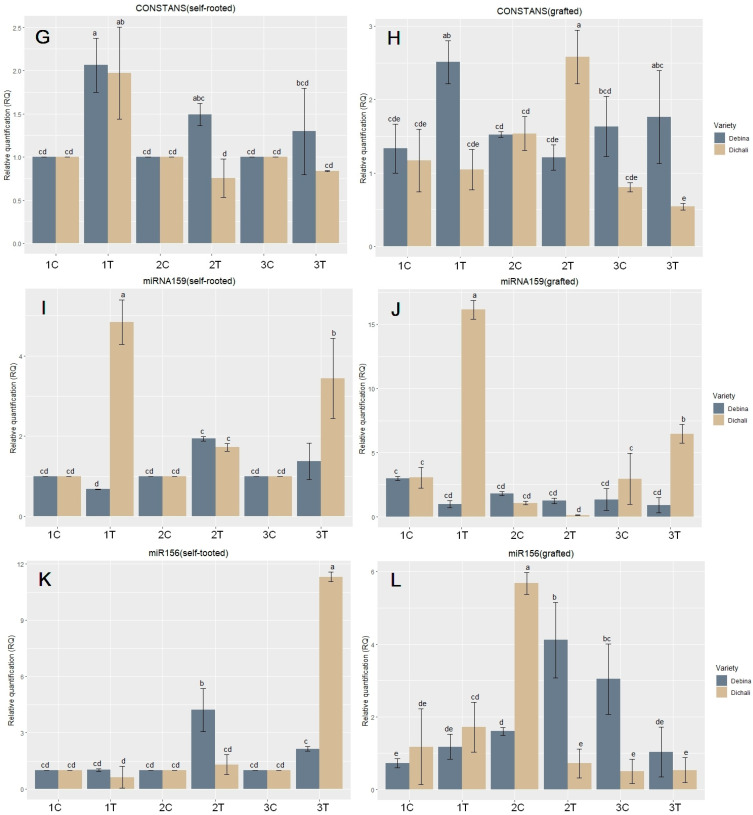
Relative expression profiles of *VvMYB101* (**A**,**Β**), *VvTOPLESS4-like* (**C**,**D**), *VvGATA26-like* (**E**,**F**) and *VvCONSTANS5-like* (**G**,**H**), *miR159* (**I**,**J**) and *miR156* (**K**,**L**) in Debina and Dichali. Self-rooted and grafted conditions are indicated. C, control; T, treatment (dehydration); 1, phase 1 (mild drought stress); 2, phase 2 (recovery); 3, phase 3 (severe drought stress). Different lowercase letters indicate differences at a significance level of 0.05.

Regarding *VvMYB101* expression, no marked changes were observed in self-rooted Debina, whereas a significant increase in transcript abundance of ~2.5-fold was evidenced in self-rooted Dichali plants under severe stress (Figure 3A). In grafted plants, a marked decrease of about ~6.5-fold was observed in severely stressed Debina but not in Dichali, whereas an increase of ~3.5-fold was shown in Dichali under mild stress (Figure 3B).

A remarkable induction of the *VvTOPLESS4-like* gene was observed upon severe dehydration conditions in both self-rooted and grafted plants in the Debina cultivar. Specifically, an increase of approximately 18-fold was observed in severely stressed self-rooted Debina plants and a marked increase of about 5-fold was evidenced in severely stressed grafted Debina plants (Figure 3C,D). Conversely, Dichali self-rooted and grafted plants did not display significant changes in *VvTOPLESS4-like* expression between control and severe dehydration stress conditions.

*GATA26-like* demonstrated a relative stability of mRNA abundance in all three phases of the experiment in both grafted and self-rooted plants (Figure 3E,F).

The *CONSTANS5-like* gene presented a slight up-regulation reflected in both self-rooted and grafted plants, especially in Debina during stress phases, but overall, the gene showed a relatively stable expression (Figure 3G,H). 

*MiRNA159* expression in both self-rooted and grafted Dichali under mild stress showed marked induction of ~5-fold, which was inversely associated with *VvTOPLESS4-like* down-regulation in those conditions (Figure 3I,J). *MiR159* expression in self-rooted and grafted Dichali under severe stress displayed an increased expression of about 4- and 2-fold (Figure 3I,J), respectively, and was anti-correlated to the respective *VvTOPLESS4-like* transcript accumulation, which remained low with no significant change. 

Regarding the expression of *miR156*, a significant increase was observed in severely stressed self-rooted plants, with a remarkable 11-fold increase in the Dichali cultivar (Figure 3K). Additionally, a significant increase (~2-fold) was evidenced in self-rooted and grafted Debina plants in the recovery phase (Figure 3K,L).

### 2.4. DNA Methylation Analysis (MSAP)

**Figure 4:**
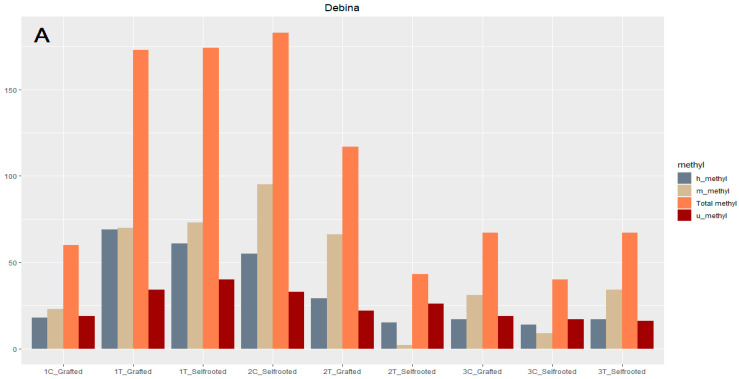

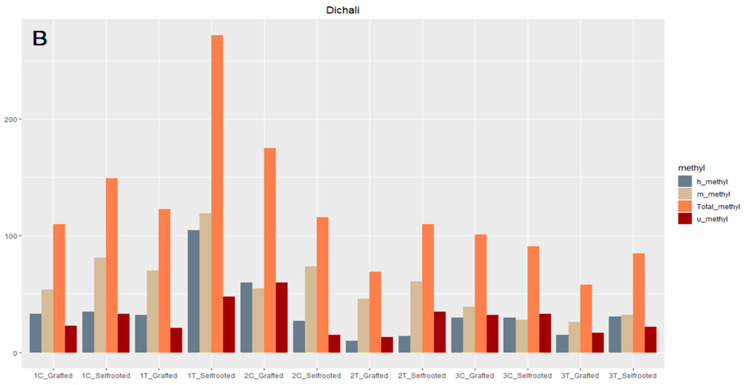
Methylation patterns (demonstrated as distinct h/m/u markers) of all samples tested (**A**,**B**). Y-axis represents total methylation (h + m alleles). h, hemimethylated; m, methylated; u, uninformative. C, control plants; T, drought stress treatment; 1, phase 1 (mild drought stress); 2, phase 2 (recovery); 3, phase 3 (severe drought stres).

The DNA methylation pattern in Debina and Dichali upon drought stress was examined by employing Methylation-Sensitive Amplification Polymorphism (MSAP) analysis and the MSAP calc_1.1 program in R software. The results are summarized in Figure 4. The data analysis did not reveal statistically significant differences among the methylation patterns (h, m, u and total markers). Nonetheless, in Debina, total DNA methylation was high in the first phase of the experiment, that is, under conditions of mild stress, whereas at the later phases of recovery and severe stress, it was profoundly decreased. In Dichali, the total DNA methylation was relatively stable at all phases of the experimental procedure; however, under severe stress, the total methylation in the stressed, grafted plants was much lower compared to the controls.

## 3. Discussion

In the current study, the response to drought conditions of two indigenous grapevine cultivars, Debina and Dichali, from the region of Epirus, was investigated at the level of physiology, gene expression, epigenetic regulation and impact of grafting onto a commercial rootstock ‘Richter 110’. Numerous studies have demonstrated that grafting substantially improves the performance of grapevine cultivars towards drought stress tolerance [55]. Grapevine is a good model plant to study drought adaptability due to the existence of large variability in drought tolerance across the Vitis species. Moreover, identifying and characterizing autochthonous cultivars with enhanced product quality and improved adaptability to a changing environment is of great importance for grapevine sustainability [56,57,58]. 

Our investigation focused on the assessment of parameters such as stem elongation, photosynthetic capacity and gas exchange and the analysis of differential expression of genes encoding transcription factors VvMYB101, VvTOPLESS4-like, VvGATA26-like, VvCONSTANS5-like, as well as conserved regulatory miRNAs, VvmiR159 and VvmiR156, in self-rooted and grafted plants during consecutive drought stress. In addition, DNA methylation analysis was performed to unravel potential drought- and graft-induced DNA methylation changes in the two grapevine cultivars.

Morphological evaluation demonstrated that in Debina, stem elongation rates severely declined upon drought stress in both self-rooted and grafted plants. Conversely, in the Dichali cultivar, a decline in growth rate was not observed upon drought stress imposition, either in self-rooted or grafted plants. The latter suggested that Dichali may be a grapevine genotype with an increased tolerance to dehydration conditions. Importantly, growth rates are maintained at a higher degree in grafted plants highlighting the positive effect of the rootstock in conferring increased drought tolerance to stressed Dichali as previously shown [59,60]. Moreover, we observed a marked enhancement in growth rates in the control, grafted plants in both cultivars, demonstrating that the Richter 110 rootstock is compatible with both Debina and Dichali genotypes and promotes plant growth.

Many studies have shown that water-deficit stress can inhibit photosynthesis, and this inhibition is related to stomatal and metabolic limitations [61,62,63]. A stomatal conductance decrease in water-stressed grapevine leaves may help reduce transpiration rates. Upon progressive dehydration conditions, stomatal closure was accompanied by a decrease in photosynthetic rate in order to maintain water balance and minimize cell damage [10,64]. Similar observations were recorded in our work for grafted Dichali and self-rooted Debina plants that were subjected to dehydration stress. It is noteworthy that the Dichali cultivar shows a more stable behavior in all photosynthetic parameters throughout the duration of the experiment in relation to the Debina cultivar. Additionally, the substantial increase in intrinsic WUE witnessed in water-stressed grafted Dichali may render this cultivar more tolerant to dehydration conditions as compared to Debina. This resembles findings from other studies where local grapevine varieties also presented better WUE compared to commercial ones under water-deficit conditions [65].

MYB family transcription factors, especially those of the R2R3-MYB subfamily, which is specific to plants, play crucial roles in plant development and the response to environmental stressors [66]. The transcription factors of the R2R3-MYB subfamily constitute key regulators in the process of responding to drought and other abiotic factors and their function (either by up- or down-regulation of specific members in a spatiotemporal manner) is associated with conferring tolerance to plants by controlling the expression of downstream stress-responsive genes [67]. Numerous studies have correlated the overexpression or down-regulation of MYB superfamily members with enhanced drought tolerance, possibly by inducing stomatal closure, increasing antioxidant enzyme activity and reducing the accumulation of ROS species [68,69,70,71,72,73,74]. 

Although extensively studied in other crops, little is known, to date, regarding MYB transcription factors and their association with drought stress responses in grapevine. In our study, a member of the *R2R3MYB* subfamily, *VvMYB101*, was investigated in terms of its expression under consecutive drought stress (mild stress–recovery–severe stress) in self-rooted and grafted plants in the two cultivars under study. Differential expression of *VvMYB101* was evidenced in response to drought stress, which depended on the grapevine cultivar and grafting. Self-rooted Debina did not display significant *VvMYB101* expression changes in response to drought stress in all three phases of the experiment. In grafted Debina, a significant reduction in *VvMYB101* expression was observed in severely stressed grafted plants. On the contrary, marked induction of *VvMYB101* was evidenced in severely stressed self-rooted Dichali and a lower induction was observed in grafted Dichali under mild stress. No significant changes were evidenced in the rest of the phases in both self-rooted and grafted plants. These observations imply a genotype-specific role for *VvMYB101* upon drought exposure and indicate an association of *MYB101* induction with drought tolerance in the Dichali grapevine cultivar. 

Noteworthy, in control, grafted plants of both cultivars, the *MYB101* gene exhibits a significant induction in all three phases, which could be attributed to the effect of the rootstock on scion gene expression during plant development along the course of the experiment. This agrees with a previous report on the effects of various rootstocks on the expression of *MYB* family members in a Pinot Noir grapevine cultivar [75]. Hence, it could be suggested that *VvMYB101* is implicated both in grapevine vegetative development and in response to water-deficit stress in a genotype and grafting-specific manner. 

The TOPLESS (TPL) family of corepressors and related proteins, altogether known as TOPLESS (TPL)/TOPLESS-RELATED (TPR), play key roles in all aspects of plant growth and development functioning as suppressors of hormonal-signaling pathways. They often interact with histone deacetylases (HDACs) leading to condensed, non-permissive chromatin configuration and subsequent transcriptional repression of TF genes [76,77]. In our study, the expression of a gene encoding a member of the *TPL* family, *VvTOPLESS4-like*, showed a remarkable induction of severe dehydration stress in both self-rooted and grafted plants in the Debina cultivar. Conversely, in Dichali, *VvTOPLESS4-like* did not display significant changes between control and severely stressed conditions both in self-rooted and grafted plants. It could be speculated that down-regulation of *VvTOPLESS4-like* in Dichali may have allowed the de-repression of important downstream stress-responsive genes and activation of tolerance-related pathways, whereas the respective pathways were suppressed by *VvTOPLESS4-like* overexpression in Debina. Nevertheless, this hypothesis awaits further experimentation.

Genes belonging to the *GATA*-gene family are involved in the activation of a series of genes related to the regulation of the response to changes in the photoperiod and additionally regulate the accumulation of chlorophyll [22]. Nevertheless, the regulatory function of these genes is affected by environmental changes, thus their expression indirectly changes under abiotic stresses, as was reported in recent studies [78,79]. In the present study, the expression of the *VvGATA26-like* gene showed no remarkable change between the stressed plants and the control plants, and only a slight up-regulation was observed in the stressed self-rooted plants of the Dichali cultivar. Based on our results, we did not monitor a significant contribution of the *VvGATA-26-like* gene to drought-stress responses in the cultivars under study.

The *CONSTANS* gene family, receiving the corresponding environmental or internal biochemical stimuli, regulates the timing and characteristics of flowering. By extension, *CONSTANS* perform a regulatory role in the photoperiodic pathway that links day length to flowering [24]. Many studies have related their expression with enhanced drought tolerance either to the regulation of flowering time [80,81] or to the manipulation of the ABA-dependent pathway [82]. In this study, however, the *VvCONSTANS5-like* gene did not show any significant difference in its expression under stress and normal conditions.

We sought to investigate miRNA-mediated epigenetic regulation of drought stress in Debina and Dichali and explore putative miRNA targets. To this end the expression of two well-conserved microRNAs, *miR159* and *miR156*, known to be involved in abiotic stress responses, was examined concomitantly with potential gene targets *VvMYB101*, *VvTOPLESS4*, *VvGATA26* and *VvCONSTANS5*, respectively [13,83]. 

*MiR159* displayed pronounced induction in mildly stressed self-rooted and grafted Dichali plants whereas a decrease was observed in Debina. Similarly, *miR159* was significantly induced both in self-rooted and grafted Dichali plants under severe stress but not in Debina. This induced *miR159* expression may be associated with the differences in drought responsiveness and enhanced stress tolerance observed in Dichali. *miR159* (as well as other miRNAs) was shown to be up-regulated under drought stress on various occasions in model and crop plants and this enhancement may lead to drought tolerance. Conversely, on other occasions, *miR159* was found to be down-regulated under drought stress or other abiotic stresses. These conflicting findings may reflect differential responses depending on plant species, genotype, developmental stage of stress imposition, severity of stress treatment and specific experimental conditions [84,85]. For example, *miR159* was up-regulated in response to drought stress in Arabidopsis, maize and barley [86,87,88] but was down-regulated in tomato, potato and cotton [89,90,91]. *miR159* was up-regulated in alfalfa and barley leaves but down-regulated in roots under drought conditions [86,92]. In addition, *miR159* was found to be down-regulated in a grapevine drought-tolerant cultivar (M4) both in leaves and roots under dehydrating conditions but did not show significant changes in a drought-sensitive cultivar [13]. In a recent study, *miR159a* was significantly up-regulated in poplar under drought stress and transgenic lines overexpressing *miR159a* exhibited reduced stomatal aperture, improved WUE and tolerance to drought [93]. Considering the above, our findings may reflect a cultivar-specific up-regulation of *miR159* in Dichali under water-deficit stress, which potentially contributes to the enhanced drought stress tolerance displayed by this grapevine cultivar.

Interestingly, the *miR159* expression pattern was inversely proportional to *VvTOPLESS4* down-regulation, evidenced under these conditions, pointing to anticorrelated expression between *VvTOPLESS4* and *VvmiR159*. Conversely, in severely stressed Debina plants, *miR159* transcript levels remained low whereas *VvTOPLESS4* was highly induced. These results agree with a previous report [13], which showed opposite trends in *VvTOPLESS4* and *VvmiR159* transcript abundance in drought-stressed genotypes, further supporting the notion of a *VvmiR159-VvTOPLESS4* (miRNA-target) regulatory module in grapevine and its involvement in drought responsiveness.

We did not observe any anticorrelated expression between *miR159* and *MYB101,* which may imply that *MYB101* is not a miR159 target, at least in these circumstances. It is possible that other members of the *R2R3MYB* family may form functional *MYB*-miR159 regulatory networks in the grapevine genotypes examined, a subject to be explored in further studies. Curiously, *miR159* expression was significantly increased in recovering (second phase) self-rooted Debina as well as Dichali plants, which may be associated with the activation of gene expression programs by *miR159*-mediated inhibition of repressing factors. 

Likewise, a pronounced increase in *miR156* expression in severely stressed self-rooted Dichali may be activating downstream gene networks and metabolic pathways associated with drought tolerance and would be in line with previous reports [51,52]. Moreover, recent studies in grapevine highlighted the involvement of *miRNA156b* up-regulation in grapevine drought tolerance [49]. Increased tolerance in self-rooted cultivars is of importance for the Epirus region since viticulturists often rely on cultivating self-rooted grapevine material, which harbors desirable organoleptic properties.

Preliminary MSAP analysis suggested a complex DNA methylation pattern that depends on cultivar and grafting status. Overall, demethylation displayed in the later phases of recovery and severe stress in Dichali might be associated with the activation of drought-responsive genes and metabolic pathways to withstand water-deficit conditions. Similar results associating a decrease in total methylation and enhanced tolerance to drought and other abiotic stresses were reported for Arabidopsis, maize, rice genotypes, ryegrass and legumes [94,95,96,97,98]. Nevertheless, our analysis should be complemented with further experiments before reaching any solid conclusions. The interplay of methylation with other mechanisms of genetic and epigenetic regulation, potentially required to establish successful drought stress responses and acquire tolerance, may be operating differently between cultivars. Recent work has suggested that the environment can have a significant impact on the methylome and that environmentally induced epigenetic changes may be the molecular basis of the ‘terroir’ effect on grapevine development and product quality. Other studies have shown that the genotype is the primary driver of DNA methylation variability [39,99,100]. Collectively, these studies suggest that the combinatorial effect of genetic/epigenetic and environmental factors may shape the performance of distinct grapevine genotypes in diverse environmental regimes.

## 4. Materials and Methods

### 4.1. Plant Material and Experimental Design

Two indigenous grapevine cultivars ‘Debina’ and ‘Dichali’ of the region of Epirus, Greece, were studied in terms of their response to drought stress in greenhouse conditions. Self-rooted plants and plants grafted on ‘Richter 110’ rootstock were grown in 10 L pots containing a commercial medium (Kronos N 50–300 mg/L, P_2_O_5_ 80–300 mg/L, K_2_O 80–300 mg/L, pH 5–6.5, salinity < 1.75 g/L) and were placed in the greenhouse (temperature range 25–30 °C) in a completely randomized block design. All the pots were normally watered and maintained in the greenhouse for one year, before the implementation of the drought stress. Ten plants for each cultivar were selected according to their uniformity of growth. Half of the plants were subjected to consecutive drought stress with an interval of 3 weeks (recovery phase) while the other half were maintained in optimal water availability conditions (control). Specifically, five self-rooted plants and five heterografts (grafted onto ‘Richter 110’ rootstock) per each cultivar were subjected to two phases of drought stress, at 50% and 25% of their pot water capacity that both lasted for 2 weeks, while between the two stress phases, there was a recovery period of 3 weeks.

### 4.2. Stem Elongation and Physiological Measurements

During the entire experiment, plant height was measured every four days. Five plants per cultivar/per treatment were used. Mature and fully developed leaves were chosen to measure the gas exchange parameters at the completion of the experiment. These measurements encompassed the determination of the net photosynthetic rate (AΝ), the concentration of carbon dioxide within the leaf’s intercellular spaces (Ci), stomatal conductance (gs) and the rate of transpiration (E). The data collection occurred during early morning, with the Li-6400XT (Li-COR, Lincoln, NE, USA) portable photosynthesis measuring system. WUE was computed at a leaf scale as the ratio between photosynthetic rate and stomatal conductance, designated intrinsic WUEi (AN/gs) [101,102]. Three leaves from each of three plants were used for gas exchange measurements. 

### 4.3. RNA Isolation and cDNA Synthesis

For gene expression analysis, young leaves from 3 individual plants/treatments were used that were harvested after the completion of each experimental phase. Leaf tissue was stored at −80 °C, until further use. Leaf samples were ground using liquid nitrogen. Total RNA was isolated via the Spectrum™ Plant Total RNA Kit (Sigma-Aldrich, St. Louis, MO, USA) and stored at −80 °C. RNA concentration and quality were estimated using NanoDrop™ ultraviolet (UV) spectrophotometry and by agarose gel electrophoresis. First-strand cDNA was synthesized from 500 ng of total RNA using the PrimeScript™ RT reagent Kit with gDNA Eraser (Perfect Real Time) of Takara Bio Inc., Shiga, Japan. The cDNA samples were then stored at −20 °C until further use. For the cDNA synthesis of miRNAs, the Mir-X miRNA First-Strand Synthesis and TB Green^®^ kit of Takara Bio Inc. was used.

### 4.4. Real-Time PCR—Relative Quantification Analysis

qPCR analysis was performed with a Step One Plus Real-Time PCR system (Applied Biosystems, Foster City, CA, USA) using SYBR Select Master Mix (Applied Biosystems, Foster City, CA, USA) according to the following protocol: 2 μL cDNA of each sample, 10 μL SYBR Select Master Mix 10×, 7.6 μL H_2_O, 0.2 μL Primer Forward, 0.2 μL Primer Reverse. Primer sequences for TF genes and corresponding miRNAs, were selected based on the study by [13]. *Actin-2* was used as an internal control for normalization. All reactions were performed in triplicate. As a reference sample, we used the self-rooted control of each experimental phase. For the micro-RNA relative quantification analysis, U6 was used as an internal control for normalization, which is a small nuclear RNA and occurs in high concentrations in species, making it the most widely used reference gene for miRNAs. The TB Green Advantage^®^ qPCR Premix kit, as well as TB Green^®^ of Takara Bio Inc. Kusatsu, Japan. The samples were loaded by the following protocol: 10 μL from TB Green Advantage qPCR Premix (2×), 0.4 μL of ROX Reference Dye LMP (50×), 0.4 μL from miRNA Primer Forward, 0.4 μL from universal miRNA Primer reverse, mRQ 3’, 6.8 μL H_2_O, 2 μL miRNA/cDNA sample. The program used for qPCR on the device was as follows: (a) 95 °C for 20 s, (b) 40 cycles in 2 stages 95 °C for 5 s, 65 °C for 34 s. Melting curves were programmed as follows: 15 s at 95 °C, 15 s at 60 °C, 20 min slow ramp and 15 s at 95 °C. Relative expression levels of all genes examined were calculated according to the 2^−∆∆Ct^ method.

### 4.5. Methylation Sensitive Amplification Polymorphism (MSAP) 

Young leaves from 3 different plants/treatments, per cultivar, were employed for the DNA methylation profiling and they were collected at the end of each experimental stage. MSAP procedure and data analysis were performed as previously described in [37]. Two aliquots of genomic DNA (200 ng each) were digested with 4U of EcoRI/3U of HpaII and 4 U of EcoRI/3U of MspI, respectively. Digestions were at 37 °C for 3 h. The resulting fragments were ligated to EcoRI and HpaII/MspI adapters at 25 °C for 3 h with 400 U/uL of T4 DNA ligase (New England Biolabs, Ipswich, MA, USA) and ligations ended by heat shock treatment at 65 °C for 10 min. The preselective PCR step was performed with a primer pair based on the sequences of the EcoRI and HpaII/MspI adapters with one additional selective nucleotide at the 3′ end (EcoRI + A and HpaII/MspI + T). Preselective PCR was performed in a total volume of 20 μL containing 1× Kapa TaqBuffer, 0.4 mM dNTPmix, 2.5 mM MgCl_2_, 30 ng of each primer EcoRI+A and HpaII/MspI+T, 1U Taq DNA polymerase (Kapa Biosystems, Wilmington, MA, USA) and 5 μL of diluted fragments (from the digestion and ligation reaction). Conditions were: 30 s at 94 °C, followed by 23 cycles of 94 °C for 30 s, 56 °C for 30 s, 72 °C for 1 min and a final hold at 72 °C for 30 min. Selective amplifications were performed in 10 μL total volume rxns containing 5 μL of diluted (1:10) pre-selective template and 0.2 mM dNTP mix, 2.5 mM MgCl_2_, 30 ng of selective HpaII/MspI primers, 30 ng of selective EcoRI primers and 1U of Taq DNA polymerase (Kapa Biosystems, Wilmington, MA, USA). Selective PCR amplification conditions were: 94 °C for 30 s, 65 °C for 30 s, 72 °C for 1 min, followed by 12 cycles of 94 °C for 30 s with Tann starting at 65 °C for 30 s, and decreasing by 0.70 °C intervals in each cycle, then 72 °C for 1 min, and finally, 22 cycles of 94 °C for 30 s, 56 °C for 30 s, 72 °C for 1 min, with a final hold at 72 °C for 30 min. Comparison of the banding patterns of EcoRI/HpaII and EcoRI/MspI reactions and analysis was performed in R software with MSAP_calc program. The MSAP experiment was conducted using 3 biological replications for each cultivar and treatment.

### 4.6. Statistical Analysis

The statistical analysis of the morphological and physiological data of the cultivars was separately performed using the *t*-test for each treatment, both in the grafted and self-rooted plants, at a significance level of 0.05. The RT-qPCR expression data were analyzed using a one-way analysis of variance (ANOVA) and Tukey’s HSD test to determine significant differential expression for transcription factors and miRNAs in the different phases of the experiment, with a level of significance of α = 0.05. The statistical analysis was performed through the R programming language (v.4.2.3) using the packages agricolae (v.1.4.0) and ggplot2 (v.3.4.2). The statistical analysis of the methylation patterns was performed using the Kruskal–Wallis test for each variety, at a significance level of 0.05.

## 5. Conclusions

In the present work, the response to consecutive drought stress of two indigenous grapevine cultivars from the Epirus region of Greece was investigated in terms of morphology, physiology, gene expression and epigenetic regulation. Our findings suggest that Dichali may be a drought-tolerant grapevine genotype and that rootstock Richter 110 potentially contributes to increased resilience to water-deficit conditions. The differential up- and down-regulation of *VvMYB101* and *VvTOPLESS4-like* transcription factor genes and *VvmiR159* and *VvmiR156* microRNAs in response to drought is dependent on cultivar and grafting status and indicates a role for these regulatory genes in the molecular mechanism of drought tolerance. Further studies on these factors will enhance our understanding of genetic and epigenetic mechanisms underlying drought stress responses in grapevines. Ultimately, these findings will be useful for incorporating genotypes with improved qualities in breeding programs and contribute to the exploitation of grapevine genetic variability towards sustainable viticulture in the context of an ever-changing environment.

## Data Availability

Data are contained within the article.
